# The Diagnosis and Therapy of XLH

**DOI:** 10.1007/s00223-025-01374-w

**Published:** 2025-04-28

**Authors:** Ineke Böckmann, Dieter Haffner

**Affiliations:** Department of Pediatric Kidney, Liver, Metabolic and Neurological Diseases, Hannover Medical School, Carl-Neuberg-Str. 1, Hannover, Germany

**Keywords:** XLH, Rickets, Osteomalacia, Burosumab, Phosphate, FGF23

## Abstract

X-linked hypophosphatemia is a rare genetic disease caused by pathogenic variants in the *PHEX* (phosphate-regulating endopeptidase homolog X-linked) gene with X-linked dominant inheritance that causes metabolic bone disease and other severe complications. PHEX dysfunction results in increased production and secretion of the phosphaturic hormone fibroblast growth factor 23 (FGF23) from bone. The consequences of FGF23 excess are renal phosphate wasting and decreased calcitriol synthesis, leading to hypophosphatemia and subsequently rickets and osteomalacia. Children with XLH usually become symptomatic in the second year of life presenting with progressive disproportionate short stature, bone pain, frontal bossing, enlarged joints, bowed legs, and a waddling gait. Various other symptoms may develop later, including dental abscesses, peritonitis, hearing loss, pseudofractures, spinal stenosis, osteoarthritis, and enthesopathies, often leading to a diminished quality of life and ultimately disability. Here, we provide an overview of the current knowledge of the pathophysiology and treatment insights of this rare and challenging disease, including the targeting of FGF23 as a therapeutic approach that has significantly improved patient outcomes.

## Introduction

X-linked hypophosphatemia (XLH) is the most common form of hereditary hypophosphatemia and caused by pathogenic variants of the *PHEX* (phosphate-regulating neutral endopeptidase homolog X-linked) gene. It has a prevalence of 1.7–4.8/100,000 children and follows an X-linked dominant inheritance with about 80% of patients having a positive family history for XLH [1–5]. The disease was first described by Albright in 1937. He reported on a patient presenting with rickets and profound hypophosphatemia not responding to high doses of vitamin D. The disease was initially called vitamin D resistant rickets, and later on X-linked hypophosphatemic rickets and eventually X-linked hypophosphatemia. Children with XLH usually present with symptoms in the first two years of life including rickets, osteomalacia, bone pain, disproportionate short stature, and later on with tooth abscesses [6, 7]. Adults with XLH are prone to additional complications, including pseudofractures, osteoarthritis, enthesopathies, spinal stenosis, obesity, and hearing loss. Due to the large spectrum of comorbidities, patients require multidisciplinary care with special attention during the transition period from pediatric to adult care.

Historically, symptomatic patients with XLH were treated with frequent oral phosphate supplements and active vitamin D which was of limited efficacy and associated with side effects, including nephrocalcinosis, hyperparathyroidism and gastrointestinal discomfort. Many features of XLH can be attributed to increased secretion of the phosphaturic hormone fibroblast growth factor 23 (FGF23) from bone [8, 9]. FGF23 excess results in renal phosphate wasting and impaired synthesis of 1,25-dihydroxyvitamin D (1,25(OH)_2_D), consecutive hypophosphatemia, and eventually in rickets and osteomalacia. This knowledge paved the way to a more specific treatment with an antibody against FGF23, i.e., burosumab [10–12]. Treatment with burosumab in pediatric XLH patients is more effective in healing rickets than treatment with phosphate salts and active vitamin D and not associated with severe side effects and has therefore become the treatment of choice for children with XLH [9]. This review describes the recent advances in the pathophysiology, diagnosis, and management of this rare disease based on the current evidence and recently updated clinical practice recommendations from European experts [6].

## Pathogenesis

X-linked hypophosphatemia is caused by pathogenic variants in the *PHEX* gene located on the short arm of the X-chromosome (p22.1–22.2), which is primarily expressed in osteoblasts, osteocytes, and odontoblasts. It has 22 exons encoding for a 749 amino acid protein [13]. There is no hot spot for pathogenic variants of the *PHEX* gene and more than 800 variants in *PHEX* were reported causing XLH which can be located in exons and introns and usually result in loss-of-function of PHEX [14]. Since females have two X-chromosomes and random X-chromosome inactivation occurs during the embryotic period, female patients with XLH are thought to show milder phenotypes than affected males, but this could not be confirmed in the majority of studies evaluating sex-dependent differences in XLH patients [15–18].

Malfunction of PHEX results in increased secretion of FGF23 from bone which binds to the FGF receptor 1-alpha-Klotho receptor-complex causing downregulation of the sodium-coupled phosphate transporters NPT2a (encoded by *SLC34A1*), and NPT2c (encoded by *SLC34A3*) and impaired synthesis of 1,25-dihydroxyvitamin D (1,25(OH)_2_D) in the proximal tubules of the kidney [19]. The result of these disturbances is renal phosphate wasting and impaired phosphate absorption from the gut as this is stimulated by active vitamin D via upregulation of the sodium-coupled phosphate transporter NaP2b (encoded by *SLC34A2*), with consecutive hypophosphatemia [1]. The latter results in rickets in children as sufficient availability of phosphate is mandatory for terminal differentiation of growth plate chondrocytes. In addition, children as well as adults with XLH develop osteomalacia due to hypophosphatemia-associated impaired bone matrix mineralization which is characterized by osteoid accumulation.

As *PHEX* encodes for a transmembrane protein with catalytic properties stemming from its extracellular domain, it was initially thought that FGF23 may be a substrate of PHEX, which was not confirmed in later studies [20]. It is thought that PHEX regulates serum FGF23 by indirect cleavage of proprotein convertases including subtilisin/kexin-type 2 (PC2) [3]. In addition, *PHEX* malfunction leads to increased synthesis of osteopontin and acid serine aspartate-rich MEPE-associated protein (ASARM) peptide in bone, which have been blamed for contributing to impaired bone matrix mineralization in XLH [21]. Studies in *Hyp* mice, an animal model of XLH, showed that excessive osteocytic FGF23 secretion results in pyrophosphate accumulation which also contributes to impaired bone mineralization [22]. Finally, FGF23 excess was shown to suppress the proliferation of growth plate chondrocytes, and therefore impair longitudinal bone growth [23, 24]. Chondrocyte columns are disorganized in *Hyp* mice, which is associated with over-activation of the ERK and MAPK signaling pathways. Of note, inhibition of the MAPK pathway, by antagonization of FGF23, resulted in improved growth plate architecture, which further supports the pathogenic role of FGF23 excess in XLH [24]. Finally, hypophosphatemia, probably in combination with a primary odontoblast defect, results in odontomalacia characterized by impaired mineralization and matrix changes in dentin and cementum [25]. Taken together, *PHEX* malfunction results in a complex osteoblast/odontoblast defect resulting in an excess of FG23 which explains its main features, i.e., rickets and osteo- and odontomalacia.

## Diagnosis

### Clinical Findings

**Fig. 1:**
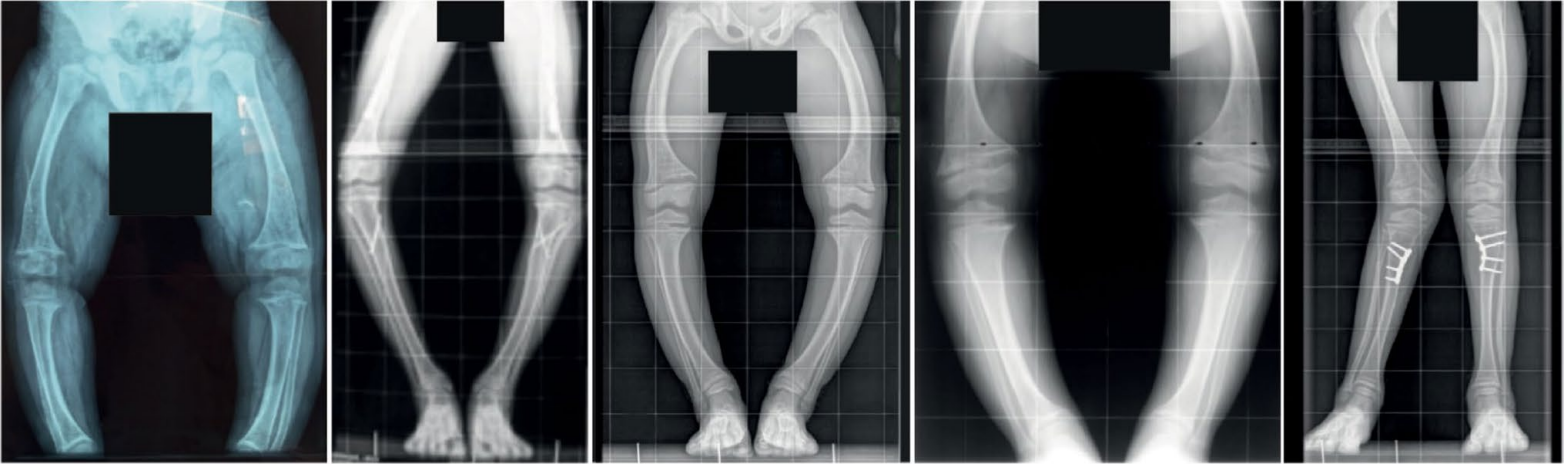
Radiographs of the lower extremities of children with X-linked hypophosphatemia. The patients show disproportionate short stature with genu varum (bowed legs). The radiographs reveal severe leg bowing, partial fraying, and irregularity of the distal femoral and proximal tibial growth plates. Note the lack of bone resorption features. Reproduced with permission from ref. [9]

**Fig. 2:**
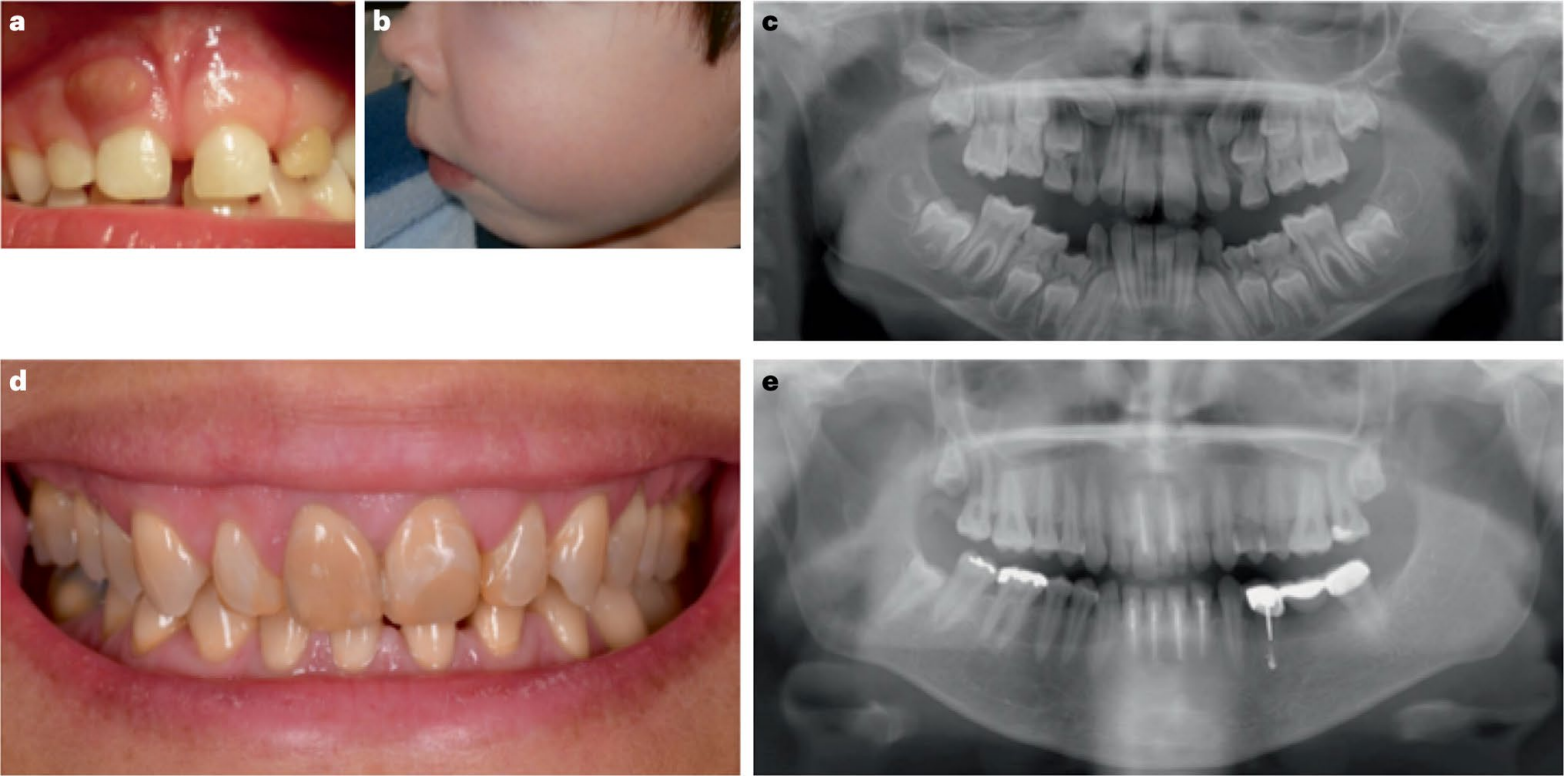
Oral manifestations of X-linked hypophosphatemia. **A** Oral clinical view of a 5-year-old male patient with XLH showing a spontaneous dental abscess on the right upper temporary central incisor. The tooth shows no discoloration or carious lesion and the child and his mother reported no history of trauma. **B** Maxillo-facial cellulitis due to spontaneous necrosis of the left upper temporary canine in the same patient at the age of 7 years. **C** Panoramic radiograph of the same patient at the age of 8 years showing mixed dentition with characteristic dental features of XLH, including a normal (slightly thin) enamel layer, a radiolucent dentin layer with enlarged pulp chambers and prominent pulp horns on both temporary and permanent teeth. **D.** Oral clinical view of a 49-year-old woman with XLH who was diagnosed at the age of 4 years. The patient was treated with oral phosphate supplements and active vitamin D during growth for 12 years before the treatment was stopped at the age of 16 years. This treatment was resumed for 4 years from the age of 40 years before being replaced with burosumab, which had been taken for 5 years. **E.** Panoramic radiograph of the same patient showing generalized horizontal alveolar bone loss and teeth treated endodontically due to dental infections. Reproduced with permission from ref. [9]

Children with XLH are normal at birth and usually become symptomatic around walking age. Clinical symptoms are similar to other forms of rickets and include growth failure, waddling gait, muscle weakness, enlarged joints, progressive lower extremity bowing (e.g., genu varum or genu valgum), and a large forehead (frontal bossing) (Fig. 1) [26]. The degree of limb deformities can be assessed by measurement of the intermalleolar and intercondylar distances [27]. Children with XLH usually develop disproportionate short stature (short legs and preserved trunk length), and may present with craniosynostosis leading to a dolichocephalic shape of the head, delayed tooth eruption, and later on with dental abscesses and sensorineural hearing loss [6, 25, 28, 29]. Spontaneous endodontic abscesses and early decay of lacteal and permanent teeth are due to impaired dentin mineralization and/or primary odontoblast defect due to PHEX malfunction (Fig. 2) [25]. In addition, patients may present with type 1 Chiari malformation and syringomyelia, which may result in headaches and neck pain, but are often asymptomatic [28]. In cases of a negative family history and/or mild clinical phenotype, diagnosis of XLH may be delayed, sometimes even until adulthood.

Adults with XLH often show disproportionate short stature, joint pain, fatigue, muscle atrophy, obesity, sleep disturbances, enthesopathies (ossification of the entheses, e.g., at the Achilles tendon), osteoarthritis (e.g., of the hip), and pseudofractures. Moreover, adult XLH patients are prone to periodontitis, alveolar bone loss, and tooth loss. These complications often result in pain, reduced quality of life, and eventually in disability [6, 30, 31].

### Radiological Findings

Rickets can be best visualized by radiography of the growth plates of rapidly growing bones, i.e., the distal ulna, knees, and ankles. The height of the physis is increased, and metaphysis may be widened, frayed or cupped (Fig. 1). Deformities of the long bone shafts can be seen, which often appear more pronounced compared to the clinical findings. In severely affected adolescents and adults, pseudofractures associated with severe osteomalacia and characterized by cortical infraction surrounded by a thickened periosteum may be detected. Interestingly, in contrast to calcipenic rickets—often resulting in intracortical bone resorption with tunneling—the cortical bone in children with XLH usually appears normal and lacks signs of bone resorption [26]. The severity of rickets can be quantified using the Rickets Severity Score (RSS) which is based on the degree of metaphyseal fraying and concavity, and the proportion of the growth plate affected at the wrist and knee [32].

### Laboratory Findings

**Fig. 3:**
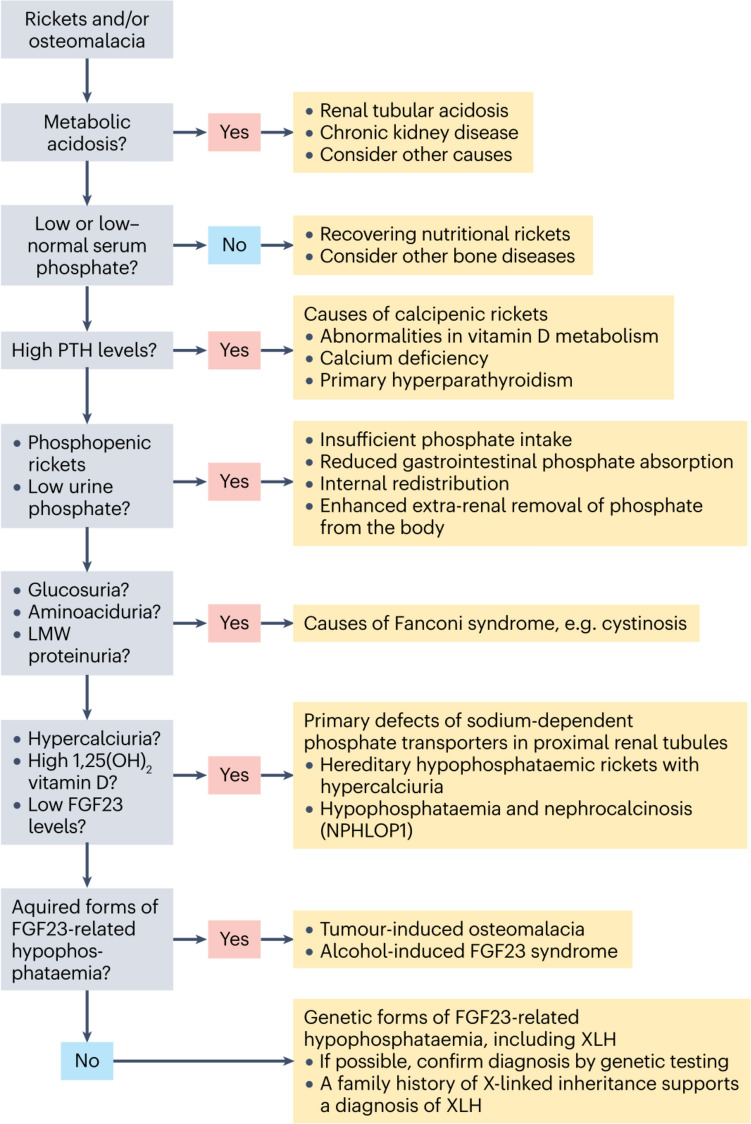
Algorithm for the diagnosis of X-linked hypophosphatemia (XLH). Patients usually present with rickets or osteomalacia and concomitant hypophosphatemia. The differential diagnosis is based on the mechanisms leading to hypophosphatemia, namely, high parathyroid hormone (PTH) activity (leading to calcipenic rickets or osteomalacia), and inadequate phosphate absorption from the gut or renal phosphate wasting (leading to phosphopenic rickets or osteomalacia). A family history of X-linked inheritance with full penetrance in female carriers strongly supports the diagnosis of XLH, which can be confirmed by genetic testing. FGF23, fibroblast growth factor 23; LMW, low molecular weight. Reproduced with permission from ref. [9]

The biochemical workup and diagnostic algorithm in patients with clinical features suggesting XLH is given in Table 1 and Fig. 3, respectively. Typical findings in patients with XLH in comparison to other forms of phosphopenic rickets and calcipenic rickets is given in Table 2. The biochemical hallmarks of XLH include hypophosphatemia, elevated alkaline phosphatase (ALP) indicating increased osteoblast activity due to rickets/osteomalacia, decreased tubular maximum reabsorption of phosphate per glomerular filtration rate (TmP/GFR) indicating renal phosphate wasting, and non-suppressed levels of intact FGF23 despite hypophosphatemia suggesting FGF23-mediated renal phosphate wasting. In contrast, serum calcium, parathyroid hormone (PTH), 25-hydroxyvitamin D levels, and calciuria are usually in the normal age-related range and patients show isolated renal phosphate wasting, i.e., no other features of renal Fanconi syndrome like glucosuria, aminoaciduria, or hypokalemia. Of note, serum phosphate levels are normal after birth in children with XLH and usually decrease below the age-related lower normal range by around 3 to 6 months of age. At this time serum, ALP concentrations (bone-specific ALP or total ALP in the absence of liver disease) start to rise as a biochemical marker of rickets/osteomalacia. Of note, non-fasting serum phosphate levels seem to be appropriate in detecting hypophosphatemia in children when using recently established pediatric reference values for serum phosphate, as these were obtained using sample collections performed throughout the day, regardless of meals [33, 34]. Serum calcium levels and urinary calcium excretion are usually normal or in the lower normal range, while hypocalcemia and hypercalciuria are not features of XLH. Parathyroid hormone levels are usually normal, but may be elevated if the patient was already started on treatment with oral phosphate supplements. Serum 25-hydroxyvitamin D levels are usually normal, and reduced 25OHD levels do not exclude the diagnosis of XLH, especially in the setting of normal PTH levels. Serum levels of 1,25(OH)_2_D are usually in the lower age-related normal range.

**Table 1:** Biochemical workup in patients with clinical features suggesting X-linked hypophosphatemia

Serum/plasma	• Phosphate (Pi), calcium, ionized calcium, albumin • Creatinine, bicarbonate • Alkaline phosphatase (ALP) • Alanine transaminase (ALT) • Aspartate transaminase (AST) • Bone specific ALP (in cases of elevated ALT/AST) • Parathyroid hormone (PTH) • 25(OH)D, and 1,25(OH)_2_D • Intact fibroblast growth factor 23 (FGF23)
Spot urine	• Dipstick: glucose, protein, pH • Potassium, sodium, calcium, Pi, creatinine, glucose, amino-acids • ß2-microglobuline (or other low molecular weight proteins)
Calculations	• Estimated glomerular filtration rate (GFR)^a^ • Urine: calcium/ creatinine ratio • Urine: phosphate/ creatinine ratio • Tubular maximum reabsorption of Pi per GFR (TmP/GFR)^a^ • Fractional tubular reabsorption of Pi (TRP)^a^

An online calculator to calculate age and/or gender-related z-scores for serum phosphate, TMP/GFR, and TRP and calcium/creatinine and phosphate/creatinine ratios in urine is available at ([42])

^a^Formulas and reference values are given in Table 3

**Table 2:** Characteristics of genetic or acquired forms of phosphopenic versus calcipenic rickets or osteomalacia

Disorder (abbreviation; OMIM#)	Gene (location)	Ca	P	ALP	U_Ca_	U_P_	TmP/GFR	FGF23	PTH	25 (OH)D^a^	1,25 (OH)_2_D	Pathogenesis
**Rickets or osteomalacia with high PTH levels (calcipenic rickets)**												
Nutritional rickets (vitamin D and/or calcium deficiency)	NA	N, ↓	N, ↓	↑↑↑	↓	Varies	↓	N	↑↑↑	↓↓, N	varies	Vitamin D/ calcium deficiency
Vitamin D-dependent rickets type 1A (VDDR1A; OMIM#264700)	*CYP27B1* (12q14.1)	↓	N, ↓	↑↑↑	↓	Varies	↓	N, ↓	↑↑↑	N	↓	Impaired synthesis of 1,25(OH)_2_D
Vitamin D-dependent rickets type 1B (VDDR1B; OMIM#600081)	*CYP2R1* (11p15.2)	↓	N, ↓	↑↑↑	↓	Varies	↓	N	↑↑↑	↓↓	varies	Impaired synthesis of 25(OH)D
Vitamin D-dependent rickets type 2A (VDDR2A; OMIM#277440)	*VDR* (12q13.11)	↓	N, ↓	↑↑↑	↓	Varies	↓	N, ↓	↑↑↑	N	↑↑	Impaired signaling of the VDR
Vitamin D-dependent rickets type 2B (VDDR2B; OMIM#164020)	*HNRNPC* (14q11.2)	↓	N,↓	↑↑↑	↓	Varies	↓	N	↑↑↑	N	↑↑	Impaired signaling of the VDR
Vitamin D-dependent rickets type 3 (VDDR3; OMIM# pending)	*CYP3A4* (7q21.1)	↓	↓	↑↑↑	↓	Varies	↓	?	↑↑↑	↓	↓	↑ inactivation of 1,25(OH)_2_D
**Phosphopenic rickets or osteomalacia due to dietary phosphate deficiency or impaired bioavailability**												
• Breastfed very low birthweight infants • Use of elemental or hypoallergenic formula diet or parental nutrition • Excessive use of phosphate binders • Gastrointestinal surgery or disorders	NA	N, ↑	↓	↑↑↑	?	↓	N^b^	N, ↓	N	N	N, ↑	Phosphate deficiency
**Phosphopenic rickets or osteomalacia with renal tubular phosphate wasting due to elevated FGF23 levels and/or signaling**												
X-linked hypophosphatemia (XLH; OMIM#307800)	*PHEX* (Xp22.1)	N	↓	↑, ↑↑	↓	↑	↓	↑, N	N, ↑^c^	N	N	↑ FGF23 expression in bone and impaired FGF23 cleavage
Autosomal dominant hypophosphatemic rickets (ADHR; OMIM#193100)	*FGF23* (12p13.3)	N	↓	↑, ↑↑	↓	↑	↓	↑, N	N, ↑^c^	N	N	FGF23 protein resistant to degradation
Autosomal recessive hypophosphatemic rickets 1 (ARHR1; OMIM#241520)	*DMP1* (4q22.1)	N	↓	↑, ↑↑	↓	↑	↓	↑, N	N, ↑^c^	N	N	↑ FGF23 expression in bone
Autosomal recessive hypophosphatemic rickets 2 (ARHR2; OMIM#613312)	ENPP1 (6q23.2)	N	↓	↑, ↑↑	↓	↑	↓	↑, N	N, ↑^c^	N	N	↑ FGF23 expression in bone
Raine syndrome associated (ARHR3; OMIM#259775)	*FAM20C* (7q22.3)	N	↓	↑, ↑↑	?	↑	↓	↑, N	N, ↑^c^	N	N	↑ FGF23 expression in bone
Fibrous dysplasia (FD; OMIM#174800)	*GNAS (*20q13.3)	N, ↓	↓	↑, ↑↑	↓	↑	↓	N, ↑	N, ↑^c^	N	N	↑ FGF23 expression in bone
Tumor induced osteomalacia (TIO)	NA	N, ↓	↓	↑, ↑↑	↓	↑	↓	N, ↑	N, ↑^c^	N	N	↑ FGF23 expression in tumoral cells
Cutaneous skeletal Hypophosphatemia syndrome (SFM; OMIM#163200) Epidermal nevus syndrome (ENS; OMIM#162900)	*RAS* (1p13.2)	N, ↓	↓	↑, ↑↑	↓	↑	↓	N, ↑	N, ↑^c^	N	N	↑ FGF23 expression due unknown mechanisms
Osteoglophonic dysplasia (OGD) (OMIM#166250)	*FGFR1* (8p11.23)	N	↓	↑, N	N	↑	↓	N, ↑	N, ↑^c^	N	N	↑ FGF23 expression in bone
Neurofibromatosis 1 (NF1; OMIM#162200)	*NF1* (17q11.2)	N	↓	↑, ↑↑	N, ↓	↑	↓	N, ↑	N, ↑^c^	N	N	↑ FGF23 expression in bone
Hypophosphatemic rickets and hyperparathyroidism (OMIM#612089)	*KLOTHO* (13q13.1)	N	↓	↑, ↑↑	↓	↑	↓	↑	↑↑	N	N	Unknown; translocation of the KLOTHO promoter
Intravenous iron therapy with ferric carboxymaltose or iron isomaltoside (‘6H-Syndrome’)^e^	NA	N, ↓	↓	↑, ↑↑	N, ↓	↑	↓	↑	↑↑	N	↓	↑ FGF23 expression in bone
**Phosphopenic rickets or osteomalacia due to primary renal tubular phosphate wasting**												
Hereditary hypophosphatemic rickets with hypercalciuria (HHRH; OMIM#241530)	*SLC34A3* (9q34.3)	N	↓	↑ (↑↑)	N, ↑	↑	↓	↓	Low N, ↓	N	↑↑	Loss of function of NPT2c in the proximal tubule
X-linked recessive hypophosphatemic rickets (OMIM#300554)	*CLCN5* (Xp11.23)	N	↓	↑ (↑↑)	N, ↑	↑	↓	varies	varies	N	↑	Loss of function of CLCN5 in the proximal tubule
Infantile hypercalcemia-2 (IH2; OMIM #616963) Hypophosphatemia and nephrocalcinosis (NPHLOP1; OMIM#612286) and Fanconi reno-tubular syndrome 2 (FRTS2; OMIM#613388)	*SLC34A1* (5q35.3)	N	↓	↑ (↑↑)	↑	↑	↓	↓	varies	N	↑	Loss of function of NPT2a in the proximal tubule
Cystinosis (OMIM#219800) and other hereditary forms of renal Fanconi syndrome	*CTNS* (17p13.2)	N, ↓	↓	↑ (↑↑)	N, ↑	↑	N, ↓	N, ↑^d^	N, ↑^d^	N	N	Cystinosin deficiency
Acquired forms of renal Fanconi syndrome	NA	N	↓	↑ (↑↑)	varies	↑	↓	↓	varies	N	↑	Drug toxicity

Ca, serum levels of calcium; P, serum levels of phosphate; ALP, alkaline phosphatase; U_Ca_, urinary calcium excretion; U_P_, urinary phosphate excretion. TmP/GFR, maximum rate of renal tubular reabsorption of phosphate *per* glomerular filtration rate; FGF23, fibroblast growth factor 23; PTH, parathyroid hormone; 1,25(OH)_2_D, 1,25-dihydroxyvitamin D; 25(OH)D, 25-hydroxy vitamin D; NA, not applicable; VDR, vitamin D receptor. ^a^Cave: prevalence of vitamin D deficiency was reported to be up to 50% in healthy children; ^b^Normal after restoration of P, but falsely reduced before restoration; ^c^PTH may be moderately elevated; ^d^Depending on the stage of chronic kidney disease. ^e^‘6H-Syndrome,’ 1**H**igh FGF23, 2**H**yperphosphaturia, 3**H**ypophosphatemia, 4**H**ypovitaminosis D, 5**H**ypocalcemia, secondary 6**H**yperparathyroidism. N, normal; ↑, elevated; ↑↑ or ↑↑↑, very elevated; ↑ (↑↑), may range widely; Table adapted from ref. [9]

Renal phosphate wasting can be proven by calculating TmP/GFR, using a second morning spot urine and serum sample taken at the same time [35–38]. The formula provided by Brodehl et al. is applicable in both the fasting and non-fasting state: TmP/GFR = P_p_—(U_p_ x P_cr_ / U_cr_), where P_p_, U_p_, P_cr_ and U_cr_ refer to serum and urine concentration of phosphate and creatinine, respectively. All values must be expressed in the same units, e.g., in milligrams per deciliter or mmol/l [35–37]. Alternatively, the nomogram from Walton and Bijvoet may be used in adults but not in children as it overestimates TmP/GFR when used in children [38, 39]. Of note, calculation of the percentage of tubular reabsorption of phosphate (TRP) is an unreliable method of evaluating renal phosphate wasting as it may be falsely normal, as it does not correct for the amount of filtered phosphate. Indeed, in cases of low serum phosphate levels, the remaining phosphate reabsorption capacity may still be enough to maintain a normal TRP, whereas TmP/GFR is already clearly reduced. Updated reference values for TmP/GFR, TRP, urinary calcium, and phosphate to creatinine ratios in children and a web calculator for TmP/GFR and age-adjusted and, where appropriate, sex-adjusted z-scores for serum phosphate TmP/GFR, TRP, and urinary calcium and phosphate to creatinine ratios for patients aged 0 to 18 years are available [34, 40–42].

Non-suppressed FGF23 concentrations in the setting of hypophosphatemia support the diagnosis of FGF23-mediated renal hypophosphatemia [9]. A 27 pg/ml cut-off value of intact FGF23 in plasma enabled discrimination between XLH and non-FGF23-related forms of renal hypophosphatemia using the Immutopics enzyme-linked immunosorbent assay (ELISA) [43]. Of note, determination of c-term FGF23 levels do not allow to discriminate between FGF23 mediated and non-mediated forms of renal hypophosphatemia. In addition, elevated intact FGF23 levels are not specific for diagnosis of XLH [9]. Finally, measurement of FGF23 levels is not available in many centers, so results are influenced by the type of test that is used, and there are pre-analytic issues which all hamper their use in clinical practice. Therefore, the diagnostic algorithm shown in Fig. 3 was designed to enable a diagnosis of XLH even if intact FGF23 concentrations cannot be determined.

### Diagnostic Approach

In children the diagnosis of XLH is based on the presence of clinical, biochemical, and/or radiological signs of rickets, impaired growth velocity and serum levels of phosphate below the age-related reference range associated with renal phosphate wasting and in the absence of vitamin D or calcium deficiency. In adults, diagnosis of XLH is based on the presence of lower limb deformities, clinical, biochemical, and/or radiological signs of osteomalacia (including pseudofractures), early osteoarthritis, spinal degeneration and stenosis, dental abscesses, and enthesopathies in the setting of reduced serum phosphate concentrations associated with renal phosphate wasting. In both children and adults, the diagnosis of XLH is further supported by a positive family history [9]. Where possible, the diagnosis of XLH should be confirmed by genetic testing.

The differential diagnosis of XLH is based on the mechanisms leading to hypophosphatemia. This includes i) high PTH activity resulting in calcipenic rickets, ii) inadequate phosphate absorption from the gut or iii) renal phosphate wasting which both result in phosphopenic rickets (Fig. 3). Renal phosphate wasting may be due to genetic or acquired tubular defects or to FGF23 excess (Table 1).

The first step in diagnosing XLH is to exclude metabolic acidosis, kidney diseases that cause non-selective tubular wasting, and causes of calcipenic rickets which are due to hyperparathyroidism (Fig. 3) [9]. The next step is to evaluate urinary calcium excretion and 1,25(OH)_2_D serum concentrations as they usually allow for distinguishing between FGF23-mediated diseases and primary tubular phosphate wasting due to defects of the sodium-dependent phosphate transporters NPT2a and NPT2c. The latter usually results in hypercalciuria and elevated 1,25(OH)_2_D serum concentrations, and suppressed PTH serum levels. The next step is to consider acquired forms of FGF23-mediated hypophosphatemia such as tumor induced osteomalacia (TIO), which should be suspected in patients manifesting after the age of two years, and those presenting with severe bone pain and a brief history of symptoms [44]. FGF23-mediated forms of hypophosphatemia can be suspected by non-suppressed FGF23 levels as outlined above. Of note, FGF23 levels are only reliable in patients prior to treatment with phosphate supplements or active vitamin D as they increase FGF23 levels. Finally, a family history of X-linked inheritance and proven genetic diagnosis of XLH in an index family member further supports the diagnosis of XLH [9]. A high-throughput sequencing approach to provide a molecular diagnosis and exclusion of other genetic forms of renal phosphate wasting is recommended as the biochemical workup does not allow to render the definitive diagnosis in the absence of a positive family history in most cases ([45]). This also allows the use of novel specific treatment for XLH as given below.

## Management

### Monitoring

Patients should be managed by multidisciplinary teams ideally at tertiary referral centers for people with XLH. Special attention should be paid to transition from pediatric to adult care. Recently published recommendations for the follow-up of patients with XLH are outlined in Table 3 [9]. This includes clinical and biochemical measures to monitor the efficacy of treatment and disease or treatment-associated (rare) complications. The most important biochemical values are serum phosphate (to assess efficacy of burosumab treatment), ALP (as a measurement of rickets/osteomalacia activity), PTH (to detect hyperparathyroidism), and calcium-to-creatinine ratio in urine (to detect hypercalciuria). Dose adjustments of treatment with phosphate and active vitamin D are based on improvement of ALP levels and of clinical and radiological signs of rickets or osteomalacia. Of note, serum phosphate concentrations are not improved by oral supplementation. In contrast, treatment with burosumab rapidly improves serum phosphate levels and TmP/GFR as a measure of renal phosphate wasting as it directly inhibits FGF23 [46, 47]. Therefore, serum phosphate and TmP/GFR are useful in monitoring the efficacy of burosumab treatment in patients with XLH. This is especially important in the first few months of therapy until other biochemical (improvement of ALP) and clinical (improvement of bone pain and skeletal deformities) signs become evident. Monitoring of complications such as nephrocalcinosis, hypercalciuria, and nephrocalcinosis are important, especially in patients on long-term treatment with phosphate salts and active vitamin D. It was also suggested to monitor 1,25(OH)_2_D serum concentrations in patients on burosumab treatment as 1,25(OH)_2_D levels may be increased above the normal range and therefore promote hypercalciuria [9]. Monitoring of FGF23 concentrations is not helpful in the management of patients with XLH as treatment with phosphate and active vitamin D stimulate FGF23 levels and burosumab interferes with the analytic assays leading to unreliable results [48, 49].

## Treatment

The availability of burosumab revolutionized the treatment of patients with XLH. A randomized clinical trial (RCT) in children with XLH aged 1–12 years showed that treatment with burosumab was superior to treatment with phosphate salts and active vitamin D over an observation time of up to 64 weeks [50]. This is true with respect to improvement of phosphate homeostasis based on serum phosphate and TmP/GFR, healing of rickets based on rickets severity score and ALP levels, improvement of lower limb deformity score, physical functioning (6-min walk test), and health-related quality of life (HRQoL). Similarly, a recent real-world study showed that HRQoL in children with XLH treated with burosumab was on an average or even slightly above that of the general population [51]. The positive results of burosumab treatment were shown to be sustained for at least 3 years [52]. Under burosumab therapy, the standardized height appeared to be stable or to improve slightly, while a real catch-up growth was rarely observed [50, 52]. These findings suggest that burosumab therapy prevents the progressive decline in standardized height that is often observed in children on treatment with phosphate salts and active vitamin D.

Although there are no RCTs comparing the efficacy and safety of burosumab treatment in symptomatic adult patients with XLH, there is evidence that burosumab treatment is superior to treatment with oral phosphate and active vitamin D with respect to improvement of clinical, biochemical, and bone histomorphometric measures of osteomalacia, including pseudofractures, phosphate homeostasis, and stiffness (as a measure of osteoarthritis), while improvements in pain and physical function did not achieve statistical significance after Hochberg multiplicity adjustment [53–57]. Overall, burosumab rapidly ameliorated bone pain and stiffness and improved fracture healing to an extent that is rarely noted with phosphate supplements and active vitamin D. Of note, the effects of burosumab treatment are not sustained after stopping treatment, suggesting that continued treatment is necessary for sustained clinical benefit [54].

Data from clinical trials and retrospective studies suggest that treatment with burosumab are of similar efficacy to prevent and treat oral manifestations including tooth abscesses and periodontitis in children and adults with XLH [47, 50, 57–60]. There is no evidence that these treatments also improve or prevent the development of hearing loss, spinal stenosis, skull base abnormalities, enthesopathies, or osteoarthritis (based on structural damage observed on X-ray). In contrast to treatment with oral phosphate and active vitamin D burosumab therapy was not associated with severe side effects, such as nephrocalcinosis, hyperparathyroidism, and hypercalciuria.

Recently, criteria were established to guide therapy in children and adults with XLH, defining a sufficient clinical response to burosumab treatment, oral phosphate, and active vitamin D (Table 3). These are applied in the following chapter.

**Table 3:** Summary of recommendations for the follow-up of patients with XLH

Examination	0–5 years	5 years to start of puberty (9–12 yrs)	Puberty	Transition to adult care	Adult	
Frequency of visits	1–3 months	3–6 months	3 months		6–12 months	
Height, weight, BMI	Every visit					
IMD and ICD**	Every visit					Yearly
Head circumference, skull shape	Every visit	Not required				
Rickets, osteomalacia, pain, stiffness, fatigue	Every visit				Every visit*	
Musculoskeletal function, 6MWT^‡^	Not feasible	Yearly		At least once	Yearly	
Orthopedic examination	Once a year in presence of substantial leg bowing			At least once	Yearly^§^	
Dental examination	Twice yearly after tooth eruption	Twice yearly		Every visit	Twice yearly	
Hearing test	Not feasible	From 8 years if symptoms of hearing difficulties				
Serum total or bone-specific ALP, calcium, phosphate, PTH, creatinine, eGFR^‡‡^	At least every 3 months	Every visit				
25(OH) vitamin D	Yearly					
Urine calcium-to-creatinine ratio^||^	Every 3 − 6 months					
Fasting (in adults) serum phosphate and TmP/GFR	Every 2 weeks during the first month, every 4 weeks during the following two months and thereafter as appropriate in patients on burosumab treatment (ideally 7–14 days after injection)					
1,25(OH)_2_ vitamin D	At least every 12 months in patients on burosumab treatment					
Blood pressure	Twice yearly					
Renal ultrasound	Every 1–2 years in patients on phosphate and active vitamin D and in patients on burosumab with pre-existing nephrocalcinosis					
Left wrist and/or lower limbs radiography	If insufficient clinical response to therapy in children; if leg bowing does not improve in growing patients; in case of short stature (bone age assessment); in case of orthopedic surgery; in case of persistent bone pain in pelvis or legs					
Dental orthopantogram and/or Cone Beam CT	Not feasible	Based on clinical need starting at the age of 6 years				
Funduscopy and brain MRI	If suspicion of cranio-synostosis, headache, and neurological symptoms	If recurrent headaches, declining school and/or cognitive performance, and/or neurological symptoms				
Spine MRI	Not required	If symptoms of spinal stenosis or persisting back pain				
Cardiac ultrasonography^¶^	In case of persistent elevated blood pressure (> 95th percentile)					
Quality of life^#^	Not feasible	Every 2 years (if available)				

IMD, intermalleolar distance; ICD, intercondylar distance; 6MWT, 6-min walk test; ALP, alkaline phosphatase; BAP, bone alkaline phosphatase; eGFR, estimated glomerular filtration rate; TmP/GFR, maximum rate of renal tubular reabsorption of phosphate normalized to glomerular filtration rate (reference values and a web calculator are given in refs [34, 41, 42], CT = computed tomography. **See refs [92] for reference values. *Also search for pseudofractures, osteoarthritis, and enthesopathy. ^‡^If available, see ref [32] for reference values. ^§^In symptomatic patients. ^‡‡^Use Schwartz formula in children, MDRD or CKD-EPI equation in adults [93]. ^||^In patients with very low urinary creatinine levels, e.g., due to low muscle mass, 24-h urine collection is recommended to assess urinary calcium excretion. The upper normal range of urinary calcium-to-creatinine ratio (mol/mol) for different age groups is as follows: < 1year, 2.2; 1–3 years, 1.4), 3–5 years, 1.1; 5–7 years, 0.8; 7–18 years, 0.7; and > 18 years, 0.57. Detailed age-related normal ranges for urinary calcium and phosphate to creatinine ratios in children are available [34]. The upper normal limit of 24 h urinary calcium excretion is 0.1 mmol (4 mg) per kg body weight for all adults or 6.2 mmol (250 mg) in female adults and 7.5 mmol (300 mg) in male adults [94]. ^¶^According to international guidelines. ^#^Using age-appropriate and disease-appropriate quality of life scales. Table adapted from ref. [9]

### Children

Based on the above-mentioned trials, a recently updated European guideline recommends burosumab as the first-line treatment in children with XLH presenting with signs of rickets including leg deformities, elevated total ALP, and/or radiological evidence of rickets [9]. If burosumab treatment is not eligible (infants aged < 1 year) or available, children with XLH should be treated with phosphate salts and active vitamin D (calcitriol or alfacalcidol). A switch from treatment with phosphate supplements and active vitamin D to burosumab should be considered in children showing insufficient skeletal response, significant adverse effects, including progressive or severe nephrocalcinosis, persistent gastrointestinal discomfort, diarrhea, hyperparathyroidism, and/or hypercalcemia, not adherent to therapy with phosphate salts and active vitamin D or persistent short stature.

The recommended burosumab starting dose is 0.8 mg/kg body weight given every 2 weeks subcutaneously, but a starting dose of 0.4 mg/kg may be sufficient as well [9]. The initial target to adapt the dosage is to raise serum phosphate levels to the lower normal range for age. The optimal time to monitor serum phosphate levels is 7–14 days after injection, and the interval of blood sampling after the last burosumab injection should be considered when assessing serum phosphate levels as they peak 7–11 days after the injection [61]. In case of hyperphosphatemia burosumab treatment should be suspended and restarted at approximately half the previous dose when serum phosphate concentration returns to below the normal range.

Clinical and biochemical measurement of rickets activity should be performed to monitor clinical response as detailed in Table 2. Assessment of radiological signs of rickets is recommended in patients showing insufficient clinical response to therapy after 12–24 months of burosumab treatment. If patients have an insufficient initial response to burosumab, the dose should be increased by steps of 0.4 mg/kg, to a maximum dosage of 2 mg/kg (maximum dose 90 mg). However, in children weighing less than 20 kg, body weight increments of 0.2 mg/kg may be more appropriate. In the case of insufficient response to burosumab (see Table 3), calcium and vitamin D deficiency with hyperparathyroidism should be excluded and treated as given below. It is recommended to keep the burosumab dose unchanged in patients showing a satisfactory response within 3–6 months even if serum phosphate levels remain below the age-related normal range. The latter is based on the finding that many patients show sufficient clinical response with normalization of serum ALP levels and improvement of bone pain and leg bowing despite persisting mild hypophosphatemia [62, 63]. The effective burosumab dose varies widely in clinical practice, ranging from 0.2 to 2 mg/kg body weight, which may at least partly be due to the fact that in contrast to clinical trials this also includes children with mild phenotypes. Adolescents with XLH generally require lower burosumab doses per body weight than children [62, 63]. With respect to transition to adult dosage it was suggested continuing burosumab treatment after the age of 18 years until at least the mid-thirties which corresponds to the time of peak bone mass. This may be helpful in ensuring optimal bone mass accrual of adequately mineralised bone in young adults in order to reduce the risk of osteoporotic fractures in adulthood [9, 64]. However, the optimal timing for switching from the pediatric to the adult burosumab dosage needs to be established.

Oral phosphate supplements should always be given in conjunction with active vitamin D to avoid hyperparathyroidism. The starting dose of phosphate salts based on elemental phosphorus is 20–60 mg (0.7–2.0 mmol) per kg body weight daily in infants and preschool children and given in 3–6 doses, with more frequent doses in younger children. The dose should be adjusted according to clinical response (improvement of rickets, growth, ALP, and PTH levels) and not exceed > 80 mg/kg daily (based on elemental phosphorus) to prevent gastrointestinal discomfort and hyperparathyroidism. In case of these adverse effects, doses of phosphate should be either decreased or the frequency of doses increased.

The recommended starting dose of calcitriol and alfacalcidol is 20–30 ng/kg body weight daily and 30–50 ng/kg body weight daily, respectively. Alternatively, patients aged at least 1 year may be started on 0.5 μg of daily calcitriol or 1 μg of daily alfacalcidol and doses adjusted on the basis of clinical and biochemical responses. Urinary calcium excretion should be kept within the normal range (Table 2) to prevent nephrocalcinosis. Additional measures may be required to decrease urinary calcium concentration, excretion, and/or crystallization by sufficient water intake, limited sodium intake and treatment with potassium citrate [9].

### Adults

Adults presenting with pseudofractures, musculoskeletal pain, stiffness, biochemical (elevated ALP), and/or radiological changes suggesting osteomalacia require medical treatment. Patients undergoing planned orthopedic surgery or dental implant surgery should also be considered to receive medical treatment. In contrast, asymptomatic adults should not be treated with oral phosphate and active vitamin D or burosumab but require careful monitoring as many adults adapt by minimizing their activities [9].

The first-line treatment in adults presenting with biochemical and/or clinical signs of osteomalacia, musculoskeletal pain or stiffness is treatment with active vitamin D in combination with phosphate salts. Treatment with burosumab is recommended in those with pseudofractures or insufficient musculoskeletal response to phosphate supplements and active vitamin D (see Table 3). Treatment with burosumab should also be considered in symptomatic patients showing adverse effects to treatment with phosphate salts and active vitamin D such as progressive nephrocalcinosis, kidney stones, hypercalcemic hyperparathyroidism, persistent gastrointestinal discomfort and/or diarrhea [9].

The recommended doses amount to 200–1,600 mg daily (based on elemental phosphorus) for phosphate, and of 0.25–0.75 daily and 0.5–1.5 μg daily when using calcitriol or alfacalcidol, respectively. A starting dose of 200–500 mg per day (based on elemental phosphorus), may be sufficient, and ideally be taken in at least three evenly spaced doses. Doses of phosphate salts and active vitamin D should be tailored according to the clinical response and presence of adverse effects [9].

The recommended starting dose of burosumab in adults is 1.0 mg/kg body weight (maximum dose of 90 mg) given every 4 weeks. Monitoring of fasting serum phosphate concentrations should be initially done between injections, ideally 7–11 days after the last injection, and after achievement of a steady state (> 2 months of a stable dosage) between injections to detect hyperphosphatemia and hypophosphatemia, respectively. In case of insufficient response (see Table 3) burosumab dose may be increased to a maximum of 90 mg, and persistent hyperparathyroidism, calcium deprivation, and non-adherence should be considered and adequately managed as potential causes [9].

Burosumab is contraindicated in pregnant women with XLH due to its reproductive toxicity demonstrated in animal studies. Therefore, symptomatic pregnant women with XLH should be considered for treatment with oral phosphate and active vitamin D. Based on studies in patients receiving other monoclonal antibodies, it is unlikely that significant amounts of burosumab are excreted in the breast milk in women on burosumab treatment [65]. In addition, burosumab is probably degraded in the gastrointestinal tract of the infant. Therefore, burosumab may be considered for treatment of symptomatic breast-feeding women with XLH [9].

### Prevention and management of hyperparathyroidism

Hyperparathyroidism is frequently noted in patients with XLH, especially in those on long-term treatment with oral phosphate and may persist after switching to burosumab treatment [62, 66]. This is thought to be due to *PHEX* deficiency, the PTH-stimulating effects of FGF23 and phosphate supplements, low 1,25(OH)_2_D levels, and/or calcium deprivation [67–74]. Hyperparathyroidism may aggravate renal phosphate wasting and therefore promote rickets and osteomalacia in XLH patients. Therefore, it is recommended to assure an adequate age-related dietary calcium intake and normal 25-OH vitamin D levels (> 20 ng/mL (50 mmol/L)), which may require supplementation with native vitamin D (cholecalciferol or ergocalciferol). Low urinary calcium excretion suggests calcium and/or vitamin D deficiency [9]. The recommended age-related daily dietary calcium intake is 200 mg (0–6 months), 280 mg (7–11 months), 450 mg (1–3 years), 800 mg (4–10 years), 1150 mg (11–17 years), 1000 mg (18–24 years), and 950 mg (> 24 years) [75, 76].

The development of high PTH levels in patients on phosphate salts and active vitamin D requires an increase of the active vitamin D dose and/or a decrease of the phosphate salts dose. Oral phosphate and active vitamin D should be reduced or stopped in case of persistent hypercalcemia and/or hypercalciuria, and patients may be switched to treatment with burosumab as this does not promote hyperparathyroidism or hypercalciuria [46, 47]. Cinacalcet is an option in patients with severe hyperparathyroidism despite normocalcemia or in those with hypercalcemic hyperparathyroidism despite the above-mentioned measures. Finally, parathyroid resection should be considered in these cases [9]. However, it was reported that hypercalcemic hyperparathyroidism persisted or recurred after parathyroid surgery in about 10% of patients with XLH [66, 77].

### Growth Hormone Treatment

Growth hormone treatment was shown to increase standardized height in short children with XLH on concomitant treatment with phosphate salts and active vitamin D for treatment periods of up to three years [66, 77]. However, final height was not increased compared to controls in a small RCT in short children treated with growth hormone, while a retrospective study reported an increase in final height by about 1.1 z-score compared to baseline height z-score in children with XLH [78, 79]. Growth hormone treatment was also shown to increase height z-score by 0.2 within 12 months in children with XLH who were switched from oral phosphate and active vitamin D to burosumab treatment [80]. However, this was an observational study and data from clinical trials on the impact of growth hormone in short children on burosumab treatment is lacking. Nevertheless, treatment with growth hormone may be an option in children with XLH showing persistent short stature despite treatment with oral phosphate and active vitamin D or burosumab [9].

### Musculoskeletal Treatment

Patients presenting with bone and joint pain, leg deformities, stiffness, muscular weakness, and reduced physical function require nonspecific measures. These patients may benefit from physiotherapy, physical activity, non-pharmacological treatment of pain, analgesics, and/or rehabilitation. This should ideally be done by involving a pain clinic to ensure multidisciplinary pain management [9].

### Orthopedic Management

Children with XLH treated with phosphate supplements and vitamin D were reported to require corrective orthopedic surgery in about one-third of patients [4, 29, 81, 82]. Burosumab was shown to be effective in substantially improving leg deformities in children with XLH with prior long-term treatment with phosphate supplements and vitamin D. Correction of leg deformities by burosumab treatment takes time, and improvements are expected to be seen after 12 months and to continue for at least 3 years [52]. Therefore, the general approach in managing leg deformities in children with XLH has changed fundamentally.

In general, patients should be encouraged to perform weight-bearing exercise, and activities to maintain joint range and to maximize strength and endurance. Physiotherapy is of special importance in patients after surgery or in those with a decreased range of movement, muscle weakness, fatigue, or instability. It is recommended to postpone surgery in growing children on burosumab, if possible, because sustained leg straightening is expected for at least 3 years after starting burosumab treatment. This allows for avoidance of unnecessary surgery or to enable less complex surgery [9].

Surgery should be considered in case of persisting deformity (mechanical axis deviation Zone 2 or greater) despite optimized medical treatment, especially when this interferes with physical function. Of note, guided growth techniques (temporary hemiepiphysiodesis) should be carried out at least 2–3 years before skeletal maturity (usually around age 14 in girls and age 16 in boys) as they depend on the remaining growth potential, whereas the risk of osteotomy-associated complications is reduced when surgery is performed at the end of growth or after skeletal maturation [9].

Osteoarthritis of large joints of the lower extremities was reported in more than 50% of young adults (< 30 years) and in up to 85% of older adults with XLH resulting in pain, stiffness, and reduced quality of life and often in disability [30, 47, 83–86]. The pathophysiology of osteoarthritis in XLH patients is complex and includes the effects of PHEX malfunction and/or altered FGF23 signaling on cartilage development and the consequences of impaired biomechanics due to rickets-associated leg deformities [87]. Patients with severe osteoarthritis should be considered for total knee arthroplasty and/or total hip arthroplasty (THA) which should be performed by surgeons with expertise in metabolic bone diseases. Cementless fixation of THA is an option in adult XLH patients [88]. Pseudofractures can be effectively treated with burosumab and do not require orthopedic surgery [9].

### Management of Oral Health

Patients with XLH should undergo close dental monitoring and may require measures, such as sealing of pits and fissures as well as supportive periodontal therapy in adults. Both treatment with burosumab or phosphate supplements and active vitamin D are effective in improving dentin and alveolar bone mineralization, and in reducing the number of tooth abscesses and severity of periodontitis in patients with XLH [47, 50, 57–60]. There is no clear evidence that either of the two available treatment options is superior to the other in terms of oral health. Therefore, XLH patients with ongoing oral manifestations, should be treated either with burosumab or active vitamin D and phosphate supplements [9]. Clinical investigations should assess the presence of pulp infection (color changes, fistula, swelling, abscess, cellulitis, or pain). Retrocoronal and/or periapical radiography or orthopantomogram may be required to detect enlarged pulp chambers and periapical bone loss. Of note, dental implant surgery in adults should be performed after at least 3 months of medical treatment and continued for 6 months thereafter [9].

### Management of Hearing Problems

Conductive and sensorineural hearing impairment was reported in adults and rarely in adolescent XLH patients [89]. In addition, XLH patients may suffer from tinnitus and/or vertigo as tabulated in Table 4.

**Table 4:** Definitions of sufficient clinical response to therapy in children and adults with XLH

Satisfactory response to therapy with oral phosphate and active vitamin D
• Children – Within 12 months: significant improvement of rickets activity, including bone pain, alkaline phosphatase (ALP) serum levels, and radiological rickets severity; ALP improves before the other parameters – Within 24 months: significant improvement of leg deformities, and normal growth velocity (> 25th percentile for sex and age) • Adults – Within 24 months: significant improvement of musculoskeletal pain, stiffness, signs of osteomalacia including radiological lesions (e.g., pseudofractures), and normalization of total or bone-specific ALP, if elevated
Satisfactory response to burosumab therapy
• Children - Within 6 months: significant improvement of renal phosphate wasting, serum phosphate levels, and rickets activity, including bone pain and ALP levels - Within 24 months: progressive improvement of leg deformities in growing children, ALP values in the age-related normal range, and normal growth velocity (> 25th percentile for sex and age) • Adults - Within 6 months: significant improvement of renal phosphate wasting, serum phosphate levels, and musculoskeletal pain - Within 12 months: improvement of musculoskeletal pain, stiffness, signs of osteomalacia including radiological lesions (e.g., pseudofractures) and total or bone-specific ALP
Insufficient response to therapy
• Insufficient response to oral phosphate and active vitamin D or to burosumab is defined in both adults and children as the lack of achievement of the above targets

No clear cut-off values for the expected changes in the indicated parameters during treatment exist, as these depend to a large extent on the severity of the disease at the start of treatment. Therapeutic decisions should be based on trends rather than individual values, taking into account all the bone health assessments mentioned above. Adapted from ref. [9]

Conductive hearing impairment is thought to be due to osteomalacia-associated petrous bone malformations. Sensorineural hearing loss is caused by changes in the composition of the endolymphatic fluid in the inner ear resulting in progressive loss of hair cells [89–91]. Therefore, XLH patients should be informed of these complications, which should generally be treated as other causes of hearing loss, with hearing aids, prevention of noise exposure, and avoidance of ototoxic drugs [9].

### Management of Neurosurgical Complications

Craniosynostosis is often asymptomatic in children with XLH and rarely requires surgery. However, careful clinical investigation, fundus examination, and brain imaging are recommended in patients presenting with neurological clinical symptoms suggestive of craniosynostosis or clinical symptoms of intracranial hypertension, lower brainstem compression, or compression of the upper cervical cord [9].

### Lifestyle Recommendations

Patients with XLH have an increased risk for obesity which is at least partly related to impaired mobility and further promoted by an inadequate caloric intake (Western diet). Measures to prevent and treat obesity as in the general population including regular physical activity are recommended in patients with XLH. All sports are allowed, provided they are not contraindicated [9].

## Conclusion

XLH is a challenging rare disease as it is associated with significant morbidity requiring life-long multidisciplinary management. Burosumab is the first-line treatment for symptomatic children with XLH, which allows normalization of phosphate homeostasis and substantial improvement in rickets in most children with XLH. Treatment with phosphate supplements and active vitamin D should be considered in adults presenting with musculoskeletal symptoms suggestive of osteomalacia. Burosumab treatment should be initiated in individuals with pseudofractures, inadequate musculoskeletal response to phosphate supplements and active vitamin D, or significant adverse events with add-on therapy.
